# High-Throughput Screen for Cell Wall Synthesis Network Module in *Mycobacterium tuberculosis* Based on Integrated Bioinformatics Strategy

**DOI:** 10.3389/fbioe.2020.00607

**Published:** 2020-06-30

**Authors:** Xizi Luo, Jiahui Pan, Qingyu Meng, Juanjuan Huang, Wenfang Wang, Nan Zhang, Guoqing Wang

**Affiliations:** ^1^Department of Pathogenobiology, The Key Laboratory of Zoonosis, Chinese Ministry of Education, College of Basic Medical Sciences, Jilin University, Changchun, China; ^2^College of Mathematics, Jilin University, Changchun, China

**Keywords:** *Mycobacterium tuberculosis*, cell wall, module, regulatory networks, enrichment analysis

## Abstract

**Background:**

*Mycobacterium tuberculosis* is one of the deadliest pathogens in humans. Co-infection of *M. tuberculosis* with HIV and the emergence of multi-drug-resistant tuberculosis (TB) constitute a serious global threat. However, no effective anti-TB drugs are available, with the exception of first-line drugs such as isoniazid. The cell wall of *M. tuberculosis*, which is primarily responsible for the lack of effective anti-TB drugs and the escape of the bacteria from host immunity, is an important drug target. The core components of the cell wall of *M. tuberculosis* are peptidoglycan, arabinogalactan, and mycotic acid. However, the functional genome and metabolic regulation pathways for the *M. tuberculosis* cell wall are still unknown. In this study, we used the biclustering algorithm integrated into cMonkey, sequence alignment, Gene Ontology (GO), Kyoto Encyclopedia of Genes and Genomes (KEGG), and other bioinformatics methods to scan the whole genome of *M. tuberculosis* as well as to identify and statistically analyze the genes related to the synthesis of the *M. tuberculosis* cell wall.

**Method:**

We performed high-throughput genome-wide screening for *M. tuberculosis* using Biocarta, KEGG, National Cancer Institute Pathway Interaction Database (NCI-PID), HumanCyc, and Reactome. We then used the Database of Origin and Registration (DOOR) established in our laboratory to classify the collection of operons for *M. tuberculosis* cell wall synthetic genes. We used the cMonkey double clustering algorithm to perform clustering analysis on the gene expression profile of *M. tuberculosis* for cell wall synthesis. Finally, we visualized the results using Cytoscape.

**Result and Conclusion:**

Through bioinformatics and statistical analyses, we identified 893 *M. tuberculosis* H37Rv cell wall synthesis genes, distributed in 20 pathways, involved in 46 different functions related to cell wall synthesis, and clustered in 386 modules. We identified important pivotal genes and proteins in the cell wall synthesis pathway such as *murA*, a class of operons containing genes involved in cell wall synthesis such as ID6951, and a class of operons indispensable for the survival of the bacteria. In addition, we found 41 co-regulatory modules for cell wall synthesis and five co-expression networks of molecular complexes involved in peptidoglycan biosynthesis, membrane transporter synthesis, and other cell wall processes.

## Introduction

*Mycobacterium tuberculosis* is considered one of the world’s most successful pathogens. The disease caused by it has been a major global health challenge (Sher et al., 2020). Since the 1950s, the discovery of first-line anti-tuberculosis (TB) drugs such as isoniazid, rifampicin, and ethambutol has effectively improved the cure rate and survival rate of TB patients. However, the emergence of multiple forms of drug-resistant strains, including a single isoniazid-resistant strain, a multi-drug-resistant strain, and a widely drug-resistant strain, has again made *M. tuberculosis* one of the leading causes of death worldwide, with a mortality of 1.5 million people in 2018 (Merker et al., 2020). Co-infection of HIV and *M. tuberculosis* increases the burden of curing TB; therefore, the development of new and effective anti-TB drugs is critical (Turner et al., 2020).

The cell wall structure of *M. tuberculosis* is unique and is extremely important for the invasion, survival, and reproduction of the bacterium in a host. The main reason for the difficulty in developing drugs for *M. tuberculosis* is that the bacterium has a hard cell wall and very low permeability. The development of *M. tuberculosis* resistance is also associated with the cell wall. Howard et al. (2018) found that *M. tuberculosis* carrying a rifampicin-resistance mutation reprograms macrophage metabolism through cell wall lipid changes. Maitra et al. (2019) described *M. tuberculosis* cell wall peptidoglycan as its fatal weakness. Thus, the cell wall of *M. tuberculosis* is an important target for the development of new anti-TB drugs.

In this study, we performed high-throughput screening of *M. tuberculosis* cell wall synthesis genes and screened key genes using bioinformatics and statistical methods to obtain new key targets for the development of anti-TB drugs.

## Materials and Methods

### Synthetic Gene Data for *M. tuberculosis* H37Rv Cell Wall

The relevant data for *M. tuberculosis* cell wall synthesis genes used in this study were obtained from the screening and integration of the following databases: TubercuList (Lew et al., 2011), TBDB (Galagan et al., 2010), PATRIC (Gillespie et al., 2011), MycoDB (Chaudhuri, 2009), GenoMycDB (Catanho et al., 2006), MyBASE (Zhu et al., 2009), MabsBase (Heydari et al., 2013), and MGDD (Vishnoi et al., 2008).

### Sequence Alignment

We used online software^1^ to compare the amino acid sequence of *M. tuberculosis* H37Rv with the amino acid sequence of *Mycobacterium smegmatis*, *Mycobacterium leprae*, *Mycobacterium bovis*, and *M. tuberculosis* H37Ra. Genes with homology greater than 60% were selected (Kuroda et al., 2001; Hellweger et al., 2014).

### Screening Essential Genes

The whole genome information for *M. tuberculosis* H37Rv was obtained from the National Center for Biotechnology Information (NCBI) and annotated using the Kyoto Encyclopedia of Genes and Genomes (KEGG) database with KEGG Orthology (KO) in accordance with the “binary relationships” provided by the KEGG Brite database. The types and functions of cell wall synthesis genes were determined using Clusters of Orthologous Groups with KO (KO COG) and the P-Score and E-Score for each KO were calculated. The E-Score was calculated with KO using the same path annotation and the P-Score was determined from the e-score. The P-Score-KEGG and P-Score-COG were also calculated based on the KEGG and COG annotations (Kong et al., 2019). These two values were in the range of 0 to 1, with 0 indicating a lack of necessity and 1 indicating necessity.

### Screening Operon Set

We applied the operon Database of Origin and Registration (DOOR) (Cao et al., 2019) established in our laboratory to classify the operon collection of cell wall genes. The DOOR database uses two prediction procedures. For operon genomes with a large number of experimental verifications, we used a non-linear classifier to train the known operon subsets based on the general characteristics of the genome and the characteristics of specific genomes. For genomes without experimental data, we used linear classification to predict operons for the general characteristics of the genome.

### Screening Co-regulatory Gene Modules

We selected all *M. tuberculosis* H37Rv gene chips in NCBI after filtering out irrelevant chip data and performed min-max normalization on each chip. We used the cMonkey double clustering algorithm to establish seed clusters (Waltman et al., 2010). We calculated the *P*-values of three such model components based on the amount of co-expressed genes, upstream sequences, and association networks. We optimized seed clusters by adding or removing related genes and proceeded to build new clusters. We used the Monte Carlo procedure to calculate the probability of each gene or condition sampled as a dual cluster gene with the conditional probability at each stage. Through these procedures, the genomic co-regulation network was identified.

### Functional Enrichment Analysis

We performed a Gene Ontology (GO) analysis of the target genes using the comprehensive database Davide^2^ for enrichment analysis, annotation, and visualization. We used the Biocarta, KEGG, National Cancer Institute Pathway Interaction Database (NCI-PID), HumanCyc, and Reactome pathway databases for pathway enrichment of the target genes. *P* < 0.05 was considered statistically significant when the threshold was ≥ two genes. We used R software and the Perl language to visualize the enrichment results. We also installed “Rcpp,” “ggplot2,” and other related software packages (Postma and Goedhart, 2019).

### Construction of Gene Regulatory Network

The protein–protein interaction (PPI) network was constructed using a gene interaction search tool database (STRING) and Cytoscape 3.6.1 was used for visualization. The Minimal Common Oncology Data Elements (MCODE), a Cytoscape network analysis plug-in for molecular complex detection, was used to deeply mine the existing modules in the network structure to find the core gene clustering modules with the highest levels of interaction.

## Results

### Statistical Analysis of Cell Wall-Related Genes in Mycobacteria

Through database annotation and sequence alignment, we screened the cell wall synthesis genes for mycobacteria. As shown in Table 1, there were 892 cell wall synthesis genes for *M. tuberculosis* H37Rv, 888 for *M. tuberculosis* H37Ra, 780 for *M. bovis*, 508 for *M. smegmatis*, and 454 for *M. leprae*.

**TABLE 1:** Cell wall synthesis network module in mycobacteria.

**Strain**	**Cell wall-related genes**	**Essential genes in cell wall**	**Operon**	**Pathway**
H37Rv	892	236	684	20
H37Ra	888	323	689	15
*M. leprae*	454	149	455	6
*M. bovis*	780	160	636	7
*M. smegmatis*	508	92	394	11

We used the operon database DOOR to assess the module distribution of cell wall synthesis genes. In *M. tuberculosis* H37Rv, 893 genes related to cell wall synthesis were located in 684 operons and 37 operons contained three or more cell wall-related genes. Multiple genes located in an operon are usually regulated by the same control region and constitute a transcription unit. The 149 genes contained in these 37 operons may be key genes that play an important role in the synthesis of the *M. tuberculosis* cell wall. There are four sets of operons, which contain more than seven genes related to the cell wall, including operons with ID numbers 7375, 7760, 6927, and 7590 displayed in the DOOR database. The ID number of the operon with the largest number of genes is 7558, up to 9. The genes *yrbE1A* and *yrbE1B* encode cell wall membrane proteins (Pasricha et al., 2011). The proteins encoded by *mce3A* and *mce3B* are not only present in the cell wall, but are also important for the virulence of *M. tuberculosis* during host invasion (Ahmad et al., 2005); 37 pairs of operons in this pathway and their details are shown in Supplementary Table S1.

The main cause of infection of the host with *M. tuberculosis* is the virulence factor. We obtained all coding genes related to virulence of TB from the VFDB database, of which 115 genes are cell wall synthesis genes. The cell wall genes that belong to virulence included the *mmpl* family which encoded cell wall lipid transporters, the cell wall mycolic acid synthase *mmA4*, and *Rv2224c* with little research and unknown specific function. Genes related to cell walls and virulence factors are shown in Supplementary Table S2.

### Function Analysis of Cell Wall-Related Genes

Essential genes are often critical for sustaining the activities of living organisms. As shown in Table 1, there are 236 essential genes related to cell wall synthesis in the whole genome of *M. tuberculosis* H37Rv. These genes are located in 161 operons, among which there are 10 operons containing more than three essential genes and five operons with more than four essential genes. Three or more operons have five or more essential genes. The six genes controlled by the operon ID6951 are all required genes that play key roles in cell wall synthesis. The six required genes include *eccA3-E3*, a member of the ESAT6 secretory system (ESX), and membrane-anchored mycosin *mycP3*. ESX secretion systems mediate various functions, participate in the metabolism of zinc and iron, and play an important role in cell wall integrity (Gaur et al., 2017).

**FIGURE 1:**
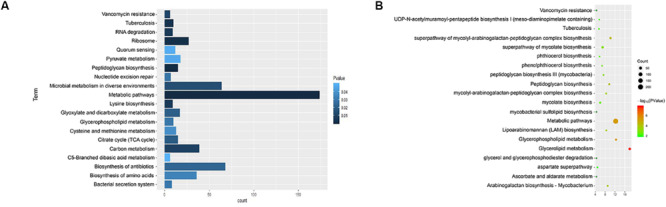
Enrichment of cell wall-related gene pathways in the standard strain of *M. tuberculosis* H37Rv. **(A)** The top 20 pathways with the lowest *p*-values (<0.05) for KEGG were selected and a histogram was created. **(B)** The top 20 pathways with the lowest *p*-values (<0.05) for Biocarta, KEGG, NCI-PID, HumanCyc, and Reactome are shown in a bubble chart.

We used KEGG, BioCyc, and Reactome pathway data to analyze cell wall synthetic genes (Figures 1A,B). The 892 cell wall synthesis genes in the H37Rv strain were distributed in 39 signaling pathways. The essential gene *murA* participates in the metabolic pathway (KEGG mtu01100), peptidoglycan biosynthesis (KEGG mtu00550), and UDP-*N*-acetylmuramoyl-pentapeptide biosynthesis I (BioCyc pwy6387). *AftB* is involved in the super pathways of mycolyl-arabinogalactan-peptidoglycan complex biosynthesis (BioCyc pwy6404) and Lipoarabinomannan biosynthesis (KEGG mtu00571). *MurA* and *aftB* are thought to be key node genes in the cell wall biosynthesis pathway. In addition, we identified some genes whose functions are currently unknown but which are located in important pathways such as the mycolyl-arabinogalactan-peptidoglycan complex biosynthesis (BioCyc PWY-6397) pathway.

**FIGURE 2:**
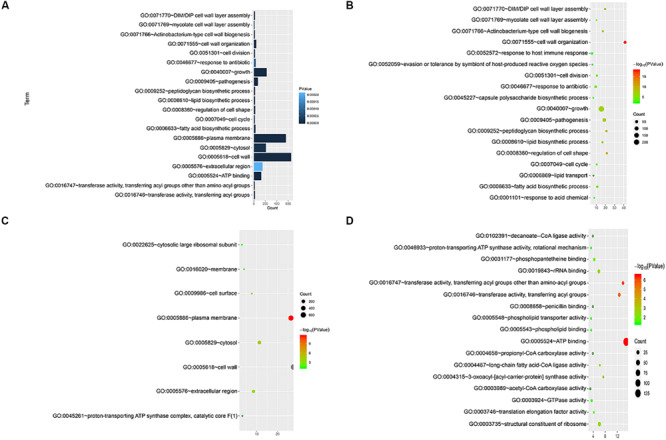
Functional significance analysis of cell wall-related genes in the standard strain of *M. tuberculosis* H37Rv. **(A)** Histogram showing the top 20 pathways identified in function analysis of cell wall-related genes. **(B–D)** Bubble charts showing the top 20 pathways in BP, CC, and MF.

We annotated the gene functions using GO and identified 46 GO items in the 892 cell wall synthesis genes. As shown in Figure 2, there were 19 items related to biological processes (BPs), nine items related to cell components (CCs), and 18 items related to molecular function (MF). The most significant BP terms were related to cell wall organization (GO:0071555), regulation of cell shape (GO:0008360), and peptidoglycan biosynthetic process (GO:0009252), as shown in Figure 2A.

**FIGURE 3:**
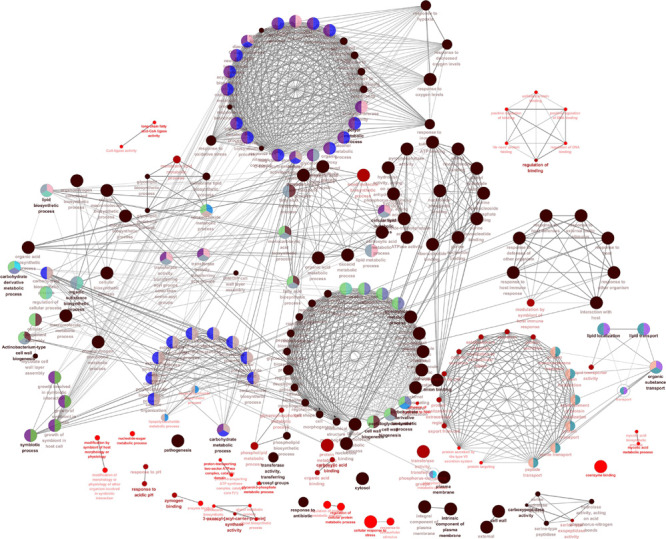
The enrichment map of GO annotation and KEGG pathway. Node size represents the number of cell wall genes expressed in specific terms. The edge thickness represents the number of genes shared by the two items connected by the edge.

We also visualized and clustered the enriched GO and KEGG terms using the cluego in Cytoscape (Figure 3). We found that most genes are enriched in important cell wall-related pathways, such as lipid biosynthetic process, peptidoglycan-base cell wall synthesis, lipid synthesis, and 3-oxoacyl-acyl-carrier-protein sythase activity. In addition, it is closely related to the pathogenicity of the host, symbiosis of the host, secretion, and pathogenesis.

### Analysis of the *M. tuberculosis* Cell Wall-Related Modules

By screening the *M. tuberculosis* cell wall modules using gene chips and the cMonkey double clustering algorithm, we found that the total number of *M. tuberculosis* modules was 600, among which 386 contained the target genes for cell wall synthesis.

Among the modules containing the target genes, 41 modules contained more than four target genes. Among these 41 modules, 16 were related to the synthesis of sugar in the cell wall, such as bicluster_0098 for the mannosyl transfer process and bicluster_0329 for the peptidoglycosyl transfer process. Fifteen modules were related to the synthesis of lipids. The modules bicluster_0068 and bicluster_0012 were related to the synthesis of mycobacterial acid (Saelens et al., 2018). There were 10 modules related to cell wall surface proteins and virulence. Among them, bicluster_0384 contained the largest number of target genes for cell wall synthesis in a single module. The nine genes contained in this module are all involved in the biosynthetic process for arabinose. For example, the *Rv0129c* coding protein plays a role in the addition of mycosyl residues in the cell wall arabinose (Jiang et al., 2020) and *Rv3806c* plays a role in the synthesis of decenyl phosphate D-arabinose (Safi et al., 2013).

**FIGURE 4:**
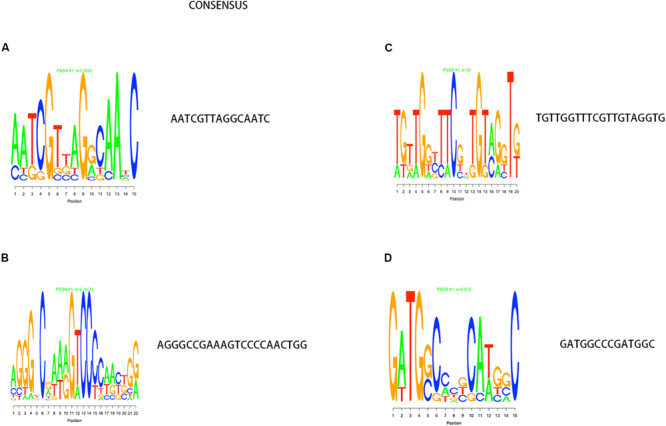
Motif analysis results with the largest number of cell wall genes in a single module cluster. **(A–D)** The motif base distribution of the four modules with the largest number of cell wall genes. In each BP base distribution in the motif, the size of the base is proportional to the corresponding frequency.

In the process of gene transcription, transcription factors complete the binding of proteins to DNA by identifying specific sequences of the double helix structure (motif). The motif is short and conservative, consisting of about 20 base pairs. Many key regulatory pathways in the cell are usually recruited by a motif (Ivarsson and Jemth, 2019). Genes located in a module are regulated by a transcription factor and have the same motif. We mapped the motif base distribution for the four modules with the largest number of cell wall genes, as shown in Figures 4A–D.

### Establishment of PPI Network and Screening of Key Genes

**FIGURE 5:**
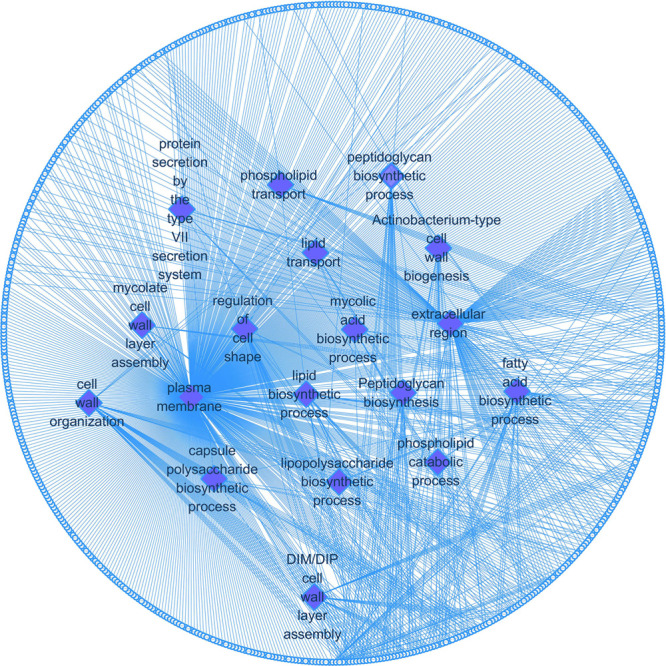
Cell wall synthesis gene and gene function regulatory network. Diamond-shaped nodes and rectangular nodes, respectively, represent gene functions and genes related to cell wall synthesis.

We enriched the function of cell wall synthesis gene and constructed the network between cell wall synthesis gene and gene function. As shown in Figure 5, the cell wall genes screened are mainly related to 18 functions, including fatty acid biosynthesis process, DIM cell wall layer assembly, and plasma membrane.

**FIGURE 6:**
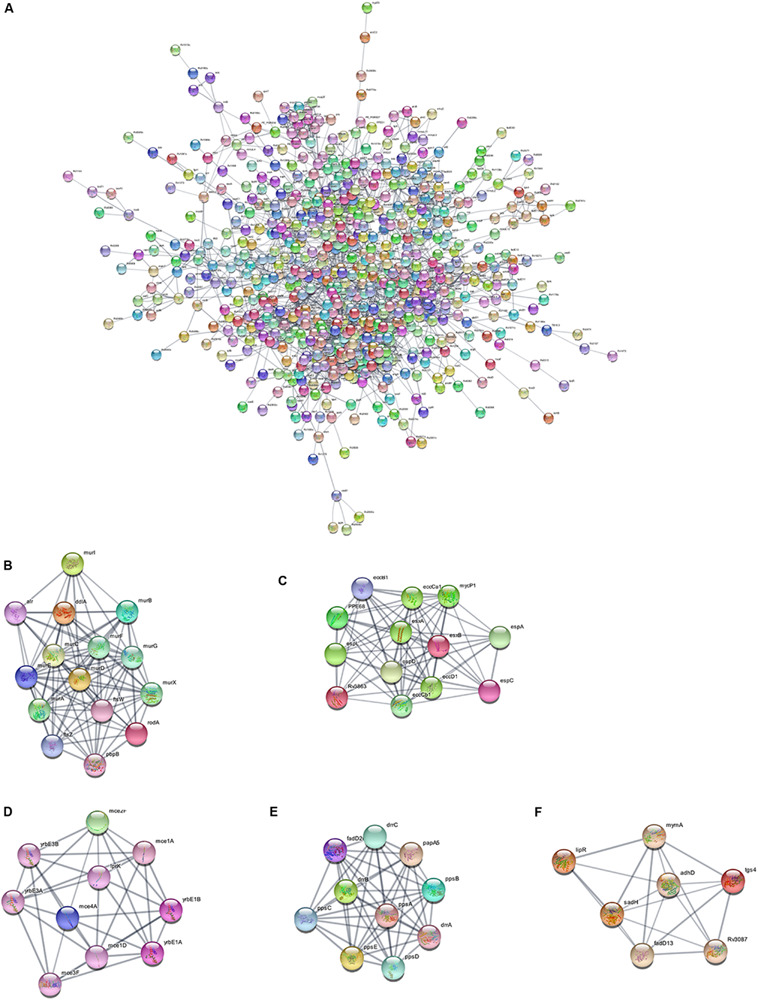
PPI network of cell wall-related genes of *M. tuberculosis*. **(A)** Protein interaction networks visualized with Cytoscape. **(B)** Molecular complex detection (MCODE) with deep excavation of the core subnet. A modular gene involved in peptidoglycan synthesis in Subnet 1. **(C)** The gene cluster for the ESX-1 secretory system. **(D)** Gene clusters encoding membrane lipid transporters. **(E)** Key gene clusters for cell wall resistance. **(F)** Fatty acids modify gene clusters.

Using the STRING database, we analyzed the interaction relationships between the cell wall synthesis genes of *M. tuberculosis* and constructed a PPI network of cell wall-related genes after deleting unconnected nodes. As shown in Figure 6A, in order to identify the key genes in the network diagram, we used MCODE to screen out five important subnets and several related genes under the condition of k-score = 2.

As shown in Figure 6B, Subnet 1 contains 14 key genes in the cell wall peptidoglycan synthesis process. Alr, the *ddla* coding protein, plays a role in the synthesis of alanine peptidoglycan (Bhat et al., 2017; Meng et al., 2019). *Ftsw*, *ftsz*, and *pbp3*-encoded proteins can form a ternary complex to potentially regulate peptidoglycan biogenesis. Roda glycosyltransferase is also involved in peptidoglycan synthesis (Wu et al., 2016). Figure 6C shows that Subnet 2 contains 13 ESX-1 secretory system-related genes. The ESX-1 secretory system is not only an important determinant of *M. tuberculosis* virulence, but is also closely related to cell wall synthesis (Wong, 2017). After elimination of the *espa* gene encoding the ESX-1 substrate, *M. tuberculosis* bacteria lose the ability to synthesize a complete cell wall structure (Chen et al., 2013). ESX-A is an early secreted antigen target that promotes the synthesis of the ESX-1 substrate and interacts with the cell membrane and cell wall of bacteria. Subnet 3 (Figure 6D) contains membrane lipid transporters. In Subnet 4 (Figure 6E), *ddrA-C* is not only the key gene in cell wall synthesis, but also the key gene for drug resistance in *M. tuberculosis* bacteria (Selvam et al., 2013). The other eight genes are related to the synthesis of lipid phthiocerol dimycocerosates (PDIM) in the cell wall. Among them, *ppsA-E* encodes the PDIM catecholic dipolyoleate (Gopal et al., 2016). All seven genes in Subnet 5 (Figure 6F) are regulated by the mymA operon and play a role in cell wall fatty acid modification (Singh et al., 2005).

**FIGURE 7:**
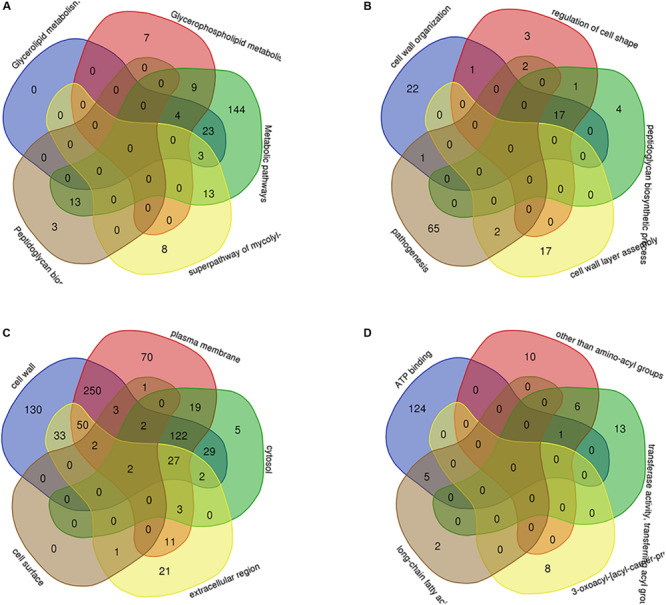
The key genes with multifunctional correlation intermingle through multiple pathways screened for the cell wall-related genes of *M. tuberculosis*. Intersecting genes in the five pathways with the most significant differences in KEGG **(A)**, BP **(B)**, CC **(C)**, and MF **(D)** are displayed in a petal diagram.

Through PPI, we identified some node genes that are crucial in cell wall biosynthesis of important sugars and lipids. In Figure 7, the petal diagram shows the genes contained in the top five annotations in BP, MF, and CC and the genes contained in the first five paths in KEGG BioCyc and Reactome. We selected the top five groups with the lowest *P*-values in all enrichments. This is to intuitively demonstrate the functional enrichment pathway for the co-regulation of key genes. The key genes for this pathway are shown in Supplementary Table S3.

## Discussion

As an important target for the development of new anti-TB drugs, the *M. tuberculosis* cell wall has attracted increasing attention. Maan and Kaur (2019) discovered *Rv2223c* in the cell wall of *M. tuberculosis*, which is a carboxyl transferase. Bothra et al. knocked out *mmpl11* and the resulting mutant strain exhibited a change in the biological activity related to mycolate wax and long-chain triacylglycerol. The knockout strain was also damaged compared to the wild strain *in vitro* granuloma model, thus demonstrating the important role of *mmpl11* in cell wall and biofilm syntheses (Bothra et al., 2018). Quigley et al. (2017) found that the expression of lipid PDIM in the cell wall of *M. tuberculosis* was negatively regulated by a novel transcription repressor, *Rv3167c*. Although extensive *M. tuberculosis* cell wall-related research has been conducted, there is still no comprehensive summary of the key genes involved in the process of cell wall synthesis.

In this study, we first screened the genes related to cell wall anabolism using multiple *M. tuberculosis* gene annotation databases. Next, we screened the essential genes for cell wall synthesis by GO functional annotation. We then evaluated the distribution of cell wall synthesis genes in the whole genome using the DOOR database established in our laboratory. Using the above methods, we obtained a lot of valuable information. For example, we identified the entire operon containing genes involved in cell wall synthesis, which is necessary for the survival of the bacterium. We employed module analysis and the cMonkey double clustering algorithm to cluster the cell wall synthesis genes. We also identified key genes by screening co-regulatory clustering modules. Through functional analysis of cell wall synthesis genes by GO and KEGG, we screened the key genes for the synthesis of important components of the cell wall, such as mycotic acid and peptidoglycan, and the key hub genes involved in multi-pathway synthesis. Finally, we created a PPI network and identified five important subnets through MCODE analysis. The intrinsic relationship between proteins in the network was used to deeply explore the genes. Molecular complexes containing key genes were extracted based on closely related regions in the PPI. Finally, we obtained the five most valuable subnets. Using Subnet 3 as an example, all genes contained in this subnet are part of the mammalian cell entry (MCE) operon (Gioffre et al., 2005). The MCE operon is present in all genera of mycobacteria and actinomycetes. However, the number of MCE operons in different strains varies, with MCE 4 in *M. tuberculosis*, MCE 3 in *M. smegmatis*, and MCE 1, 2, and 4 in *M. bovis*. It is unknown why the MCE 3 operon is absent from *M. bovis* (Kumar et al., 2005). The MCE operons help *M. tuberculosis* ingest cholesterol in the host to keep the bacteria alive. Lack of the MCE operon causes a serious imbalance of lipid content in the *M. tuberculosis* cell wall. Sally et al. reported free mycolic acid accumulation in the cell wall of the MCE 1 operon mutant strain of *M. tuberculosis* (Singh et al., 2018). However, the genes contained in Subnet 4, such as *ppsa* and *ppsb*, were significantly altered in drug-resistant bacteria (Cantrell et al., 2013). We believe that *ppsa* changes the expression of PDIM in the cell wall by changing the approach of the multi-subunit non-iterated polyketide synthase system (Vergnolle et al., 2015). This makes the bacterial cell wall thicker and causes bacterial drug efflux. We used bioinformatics and statistical methods to comprehensively scan all the genes synthesized in the *M. tuberculosis* cell wall and to screen out new targets that can be used as new anti-*M. tuberculous* cell wall targeting drugs.

## Data Availability Statement

All datasets presented in this study are included in the article/Supplementary Material.

## Author Contributions

GW designed this study. XL and JP wrote the manuscript. XL analyzed the data. QM, WW, JH, and NZ contributed in the data collection. All authors contributed to the article and approved the submitted version.

## Conflict of Interest

The authors declare that the research was conducted in the absence of any commercial or financial relationships that could be construed as a potential conflict of interest.

## Footnotes

1 www.ncbi.nlm.nih.gov/blast/

2 https://david.ncifcrf.gov/
